# Portal Vein Thrombosis and Hepatic Infarction as a Rare Complication of Disseminated Tuberculosis in Advanced HIV Disease: A Case Report From Mthatha Regional Hospital, South Africa

**DOI:** 10.7759/cureus.117094

**Published:** 2026-09-28

**Authors:** Lithalethu Nkomonye, Lusanda Zongo, Sisipho Mgumane, Ramprakash Kaswa

**Affiliations:** 1 Internal Medicine, Mthatha Regional Hospital, Mthatha, ZAF; 2 General Surgery, Mthatha Regional Hospital, Mthatha, ZAF; 3 Family Medicine / Internal Medicine, Walter Sisulu University, Mthatha, ZAF

**Keywords:** ascites, disseminated tuberculosis, hepatic infarction, hiv, hypercoagulability, portal vein thrombosis, tb

## Abstract

Portal vein thrombosis (PVT) is an uncommon yet potentially life-threatening vascular complication defined by partial or complete obstruction of portal venous flow. Active tuberculosis (TB) is increasingly recognised as a prothrombotic condition, with systemic inflammation, endothelial dysfunction, platelet activation, and alterations in natural anticoagulant pathways contributing to thrombus formation. The risk of PVT may be further increased in the context of advanced human immunodeficiency virus (HIV) infection. Nevertheless, reports of PVT associated with disseminated TB in patients with advanced HIV disease remain rare, particularly in high-burden, resource-limited settings.

A 26-year-old woman with advanced HIV infection and poor adherence to antiretroviral therapy presented with progressive abdominal distension, abdominal pain, constitutional symptoms, jaundice, and massive ascites. Laboratory investigations revealed severe immunosuppression (CD4 count 45 cells/µL), pancytopenia, transaminitis, coagulopathy, and marked hypoalbuminaemia. GeneXpert testing of ascitic fluid and sputum confirmed rifampicin-sensitive *Mycobacterium tuberculosis*. Contrast-enhanced computed tomography identified extensive PVT involving the main and right portal veins, with collateral flow and wedge-shaped hypoattenuation of the right hepatic lobe, consistent with hepatic infarction. Additional findings included extensive abdominal lymphadenopathy and splenic lesions. Chest imaging showed miliary nodular infiltrates and left lower lobe consolidation, supporting a diagnosis of disseminated tuberculosis. The superior mesenteric vein and artery remained patent.

The coexistence of advanced HIV infection and disseminated TB provides a plausible multifactorial basis for PVT, resulting from synergistic effects of persistent systemic inflammation, immune dysfunction, activation of endothelial and coagulation pathways, and local impairment of portal venous flow due to extensive tuberculous lymphadenopathy. Hepatic infarction is an uncommon complication of extensive PVT, given the liver's dual arterial and portal blood supply. Management of TB-associated PVT is particularly challenging in the presence of thrombocytopenia, coagulopathy, and advanced hepatic dysfunction, as these factors significantly increase bleeding risk and complicate anticoagulation decisions.

PVT should be considered in patients with advanced HIV disease and disseminated TB who present with abdominal pain, ascites, or features of portal hypertension. This case demonstrates the rare association of disseminated TB with extensive PVT and hepatic infarction in advanced HIV disease, emphasising the interplay between systemic TB-associated inflammation, HIV-related prothrombotic mechanisms, and local vascular compromise. Early recognition, appropriate cross-sectional imaging, microbiological confirmation, and multidisciplinary assessment are critical for timely diagnosis and management of this potentially life-threatening complication.

## Introduction

Portal vein thrombosis (PVT) is characterised by thrombotic occlusion of the portal venous system and may occur in association with cirrhosis, malignancy, abdominal infections, inflammatory disorders, or inherited thrombophilias [1]. Although uncommon, PVT can lead to portal hypertension, intestinal ischaemia, hepatic infarction, and significant morbidity if not recognised promptly [2].

Tuberculosis remains a major global health challenge, particularly in sub-Saharan Africa, where human immunodeficiency virus (HIV) co-infection is prevalent. Increasing evidence suggests that tuberculosis induces a hypercoagulable state through endothelial injury, increased pro-inflammatory cytokine production, elevated fibrinogen levels, platelet activation, and reductions in natural anticoagulants such as antithrombin III and protein C [3,4]. HIV infection further amplifies thrombotic risk through chronic immune activation, endothelial dysfunction, and opportunistic infections [5].

Although venous thromboembolism is increasingly recognised in tuberculosis, involvement of the portal venous system remains rare [6]. We present a case of PVT with hepatic infarction and massive ascites in a young woman with advanced HIV disease and disseminated tuberculosis.

## Case presentation

A 26-year-old woman with a six-year history of HIV infection presented to Mthatha Regional Hospital with progressive abdominal distension persisting for over one month. Associated symptoms included generalised malaise, fatigue, unintentional weight loss, facial puffiness, scleral icterus, right upper quadrant and epigastric pain, early satiety, intermittent nausea and vomiting, and shortness of breath. She was receiving antiretroviral therapy consisting of tenofovir, lamivudine, and dolutegravir (TLD); however, poor adherence to treatment had been documented during the preceding two years (Table 1).

**Table 1:** Summary of Laboratory Investigations on Admission FBC: Pancytopenia was present, characterised by leukopenia, anaemia, and thrombocytopenia.

Investigation	Result	Reference Range	Interpretation
CD4 Count	45 cells/µL	500–1500 cells/µL	Severe immunosuppression
HIV Viral Load	968,876 copies/mL	Undetectable (<50 copies/mL)	Uncontrolled HIV infection
White Cell Count	3.3 ×10⁹/L	4.0–11.0 ×10⁹/L	Leukopenia
Haemoglobin	7.1 g/dL	12.0–16.0 g/dL	Severe anaemia
Platelet Count	67 ×10⁹/L	150–400 ×10⁹/L	Thrombocytopenia
Aspartate Aminotransferase (AST)	108 U/L	<40 U/L	Elevated
Alanine Aminotransferase (ALT)	77 U/L	<40 U/L	Elevated
Albumin	6 g/L	35–50 g/L	Severe hypoalbuminaemia
International Normalised Ratio (INR)	2.0	0.8–1.2	Coagulopathy

She denied experiencing fever, night sweats, haematemesis, melaena, alcohol consumption, or use of traditional herbal medications. On examination, she appeared chronically ill and exhibited marked abdominal distension with shifting dullness, consistent with massive ascites (Table 2). There was associated right upper quadrant tenderness, although neither hepatomegaly nor splenomegaly was clinically appreciable.

**Table 2:** Ascitic Fluid Analysis Interpretation: Although the ascitic fluid ADA level was not markedly elevated, GeneXpert MTB/RIF detected rifampicin-sensitive *Mycobacterium tuberculosis*, confirming tuberculous involvement of the peritoneum. In the setting of advanced HIV infection and severe immunosuppression (CD4 count 45 cells/µL), lower-than-expected ADA levels may occur and should not exclude the diagnosis of abdominal tuberculosis.

Parameter	Result	Clinical Significance
Total Protein	20 g/L	Suggestive of an inflammatory or infectious process
Lactate Dehydrogenase (LDH)	270 U/L	Elevated, supporting active inflammation
Adenosine Deaminase (ADA)	14.5 U/L	Below the conventional diagnostic threshold for tuberculous peritonitis; however, a low ADA does not exclude tuberculosis in severely immunocompromised patients
Bacterial Culture	No growth	No evidence of bacterial peritonitis
GeneXpert *Mycobacterium tuberculosis*/Rifampicin sensitive (MTB/RIF)	Positive	Confirmed *Mycobacterium tuberculosis* with no rifampicin resistance detected

Investigations

Abdominal Ultrasound

Abdominal ultrasonography revealed significant ascites, multiple enlarged para-pancreatic and splenic hilar lymph nodes, and numerous small hypoechoic lesions within the spleen. The liver was normal in size, exhibited mildly increased echogenicity, and showed no focal hepatic lesions. Both the gallbladder and pancreas appeared unremarkable.

Computed Tomography (CT) Abdomen

Contrast-enhanced computed tomography (CECT) of the abdomen revealed extensive PVT accompanied by right hepatic lobe infarction. Additionally, massive ascites, abdominal lymphadenopathy, and splenomegaly were observed, with the spleen exhibiting heterogeneous parenchymal enhancement and multiple hypodense lesions (Figure 1).

**Figure 1:**
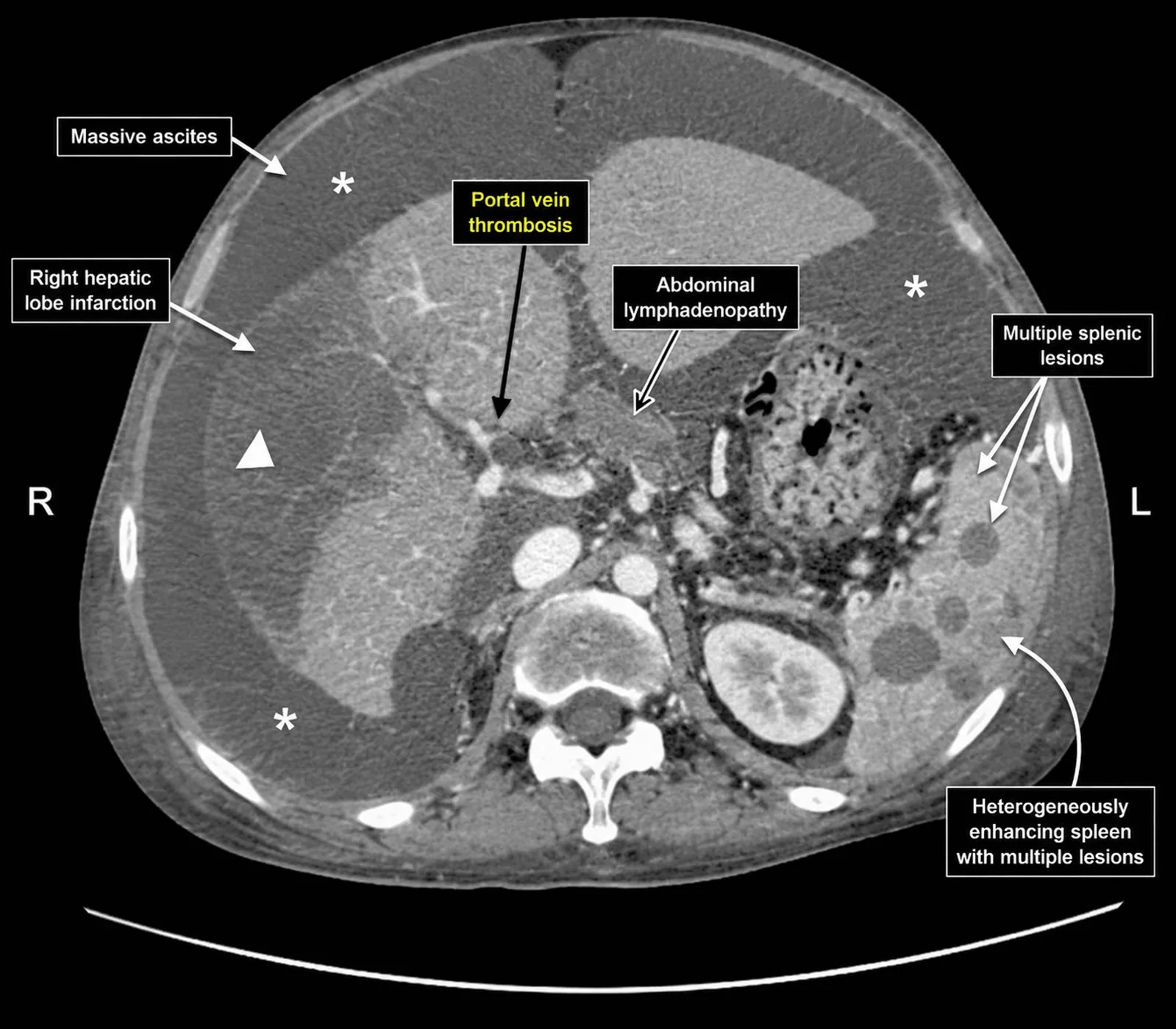
Contrast-enhanced CT of the abdomen demonstrating portal vein thrombosis with hepatic and splenic involvement.

Chest X-ray Imaging

Thoracic imaging demonstrated diffuse bilateral miliary nodular infiltrates with left lower lobe consolidation, findings consistent with disseminated pulmonary tuberculosis (Figure 2).

**Figure 2:**
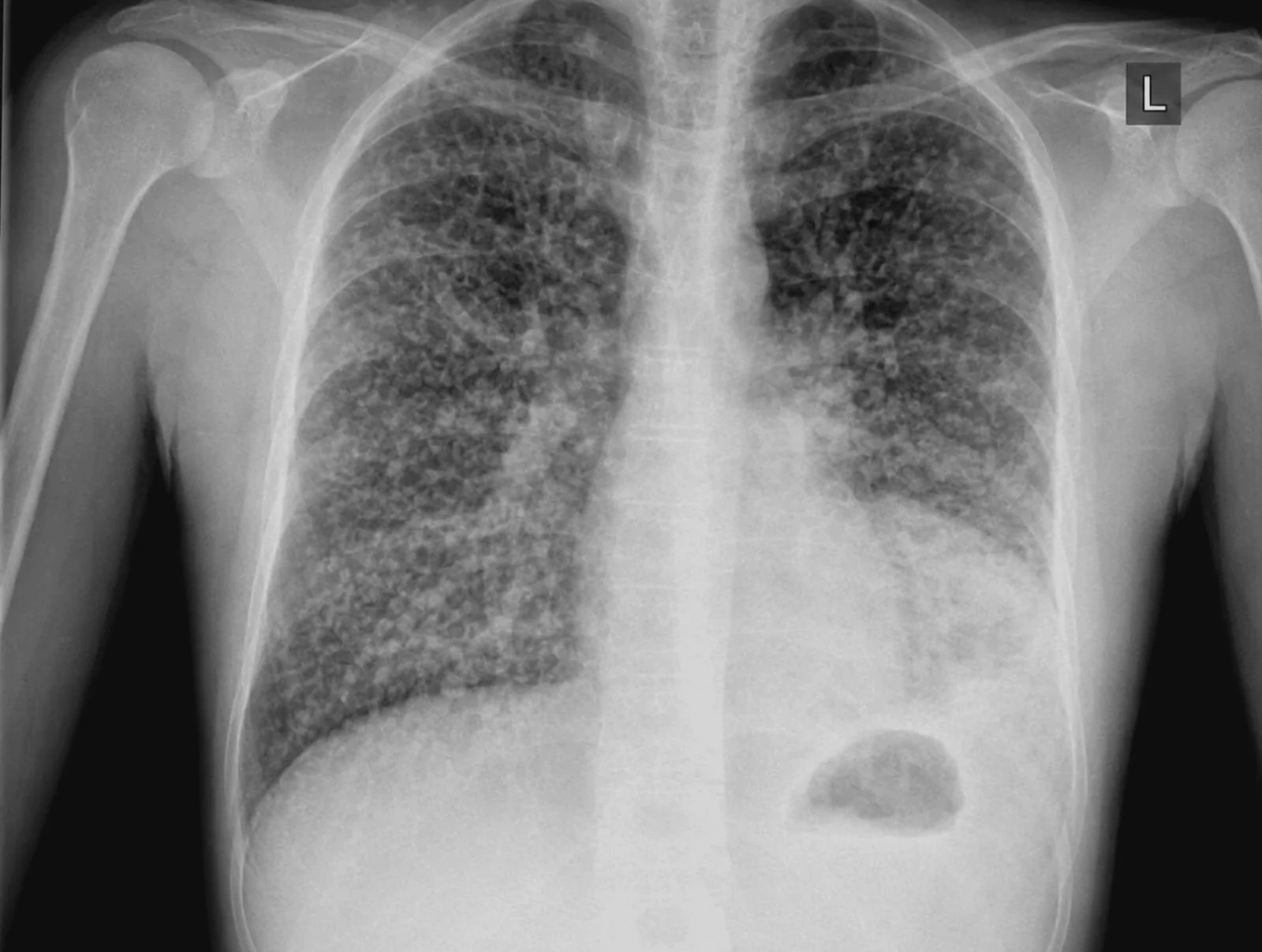
Chest radiograph demonstrating miliary tuberculosis.

Diagnosis

The patient presented with advanced HIV infection characterised by severe immunosuppression and disseminated tuberculosis affecting the lungs, peritoneum, abdominal lymph nodes, and spleen. The clinical course was complicated by PVT with right hepatic lobe infarction, massive ascites secondary to abdominal tuberculosis and portal hypertension, pancytopenia, and severe hypoalbuminaemia (Tables 1, 2).

Management

Upon admission, all chronic medications were temporarily withheld due to the patient's acute clinical status. Initial management involved correction of coagulopathy with vitamin K and administration of lactulose for prophylaxis of hepatic encephalopathy, pending completion of further diagnostic investigations.

After radiological confirmation of PVT, therapeutic anticoagulation with warfarin was initiated following multidisciplinary evaluation of risks and benefits, considering thrombocytopenia, coagulopathy, severe hypoalbuminaemia, and hepatic infarction. Anticoagulation was titrated based on the international normalised ratio (INR), with regular monitoring to maintain therapeutic levels.

Following confirmation of rifampicin-sensitive tuberculosis, standard first-line anti-tuberculosis therapy (rifampicin, isoniazid, pyrazinamide, and ethambutol) was commenced promptly. Antiretroviral therapy (ART) was deferred for two weeks to reduce the risk of immune reconstitution inflammatory syndrome (IRIS) and was successfully reintroduced once the patient had clinically stabilised.

Supportive management comprised nutritional rehabilitation, serial large-volume therapeutic paracenteses with intravenous human albumin replacement, and initiation of spironolactone, furosemide, and carvedilol to manage portal hypertension and ascites.

The patient demonstrated progressive clinical improvement, achieving complete resolution of ascites by discharge. At follow-up, clinical stability was maintained on anti-tuberculosis therapy, ART, warfarin, and medical therapy for portal hypertension, with continued INR monitoring and dose adjustments as necessary.

Clinical outcome and follow-up

After initiation of anti-tuberculosis therapy, anticoagulant therapy, and supportive management, the patient demonstrated gradual clinical improvement. Abdominal distension subsided, and constitutional symptoms resolved. The patient was discharged after 56 days of hospitalisation with scheduled follow-up at the MOPD MRH.

At four weeks, eight weeks, and three months follow-up, the patient reported improved appetite and functional status. Subsequent clinical assessments indicated reduced ascites and enhanced symptom control. The patient maintained adherence to both antiretroviral and anti-tuberculosis therapy.

## Discussion

Tuberculosis is increasingly recognised as a systemic prothrombotic disorder. Elevated levels of tumour necrosis factor-alpha, interleukin-6, fibrinogen, factor VIII, and platelet activation contribute to thrombus formation during active disease [3,4]. Simultaneously, reductions in antithrombin III, protein C, and protein S may further predispose patients to venous thrombosis [7].

Individuals living with HIV exhibit a substantially increased risk of thromboembolic disease compared with the general population. Contributing mechanisms include chronic inflammation, endothelial dysfunction, opportunistic infections, malignancy, antiphospholipid antibodies, and severe immunosuppression [5,8]. In this case, both advanced HIV infection and disseminated tuberculosis likely acted synergistically to promote thrombosis. Non-adherence to antiretroviral therapy may have further contributed to this prothrombotic state by permitting persistent HIV viraemia, progressive immunosuppression, and sustained immune activation. Uncontrolled HIV infection is associated with endothelial dysfunction, persistent inflammation, and activation of coagulation pathways, all of which may increase the risk of thromboembolic disease [9-11]. Advanced immunosuppression also increases susceptibility to opportunistic infections such as disseminated tuberculosis, which may further amplify systemic inflammation and coagulation activation [5,8]. Previous reports of PVT in people living with HIV have described profound immunodeficiency, disseminated mycobacterial infection, and abdominal lymphadenopathy as potential contributing factors [12]. Therefore, in the present case, inadequate HIV control may have indirectly amplified the thrombotic risk through the combined effects of persistent viral inflammation, severe immunosuppression, and disseminated tuberculosis. However, a direct causal relationship between antiretroviral therapy non-adherence and PVT cannot be established.

PVT represents an uncommon manifestation of tuberculosis. Proposed mechanisms include direct vascular compression by tuberculous lymphadenopathy, local inflammatory extension, and systemic hypercoagulability [6,13]. The extensive abdominal lymphadenopathy observed in this patient may have contributed to impaired portal venous flow and subsequent thrombus formation.

The role of anticoagulation in tuberculosis-associated PVT remains incompletely defined. Current evidence supports anticoagulation in acute PVT to promote recanalisation and prevent thrombus extension; however, treatment decisions should be individualised for patients with significant thrombocytopenia, coagulopathy, or advanced hepatic dysfunction [14,15]. In this case, the substantial bleeding risk presented a therapeutic challenge and necessitated careful multidisciplinary assessment.

The occurrence of hepatic infarction is particularly significant. Owing to its dual blood supply from the hepatic artery and portal vein, the liver is generally protected against ischaemic injury. Hepatic infarction therefore typically indicates severe vascular compromise and has been reported only rarely in association with PVT [14]. In this case, a filling defect in the main portal vein extended to the right portal vein, with collateral flow, and was accompanied by wedge-shaped hypoattenuation of the right lobe of the liver. The superior mesenteric vein (SMV) and superior mesenteric artery (SMA) remained patent. These findings support portal venous compromise as a likely contributor to the hepatic ischaemic changes, particularly in the context of extensive PVT and the absence of SMV or SMA occlusion.

This case further underscores the diagnostic challenges associated with massive ascites in young HIV-positive individuals. Although cirrhosis remains the most common cause of portal hypertension globally, infectious causes such as disseminated tuberculosis must be actively considered in endemic regions. Early imaging, microbiological confirmation, and multidisciplinary management are essential to improving outcomes.

## Conclusions

PVT should be considered in patients with advanced HIV disease and disseminated tuberculosis who present with abdominal pain, ascites, or other features indicative of portal hypertension. This case demonstrates the rare occurrence of PVT associated with disseminated tuberculosis in a patient with advanced HIV disease, with hepatic infarction as an additional uncommon complication. The report emphasises the complex interactions among HIV-associated immune dysfunction, tuberculosis-induced systemic inflammation, and a prothrombotic state. Early recognition, appropriate imaging, and microbiological confirmation are critical for timely diagnosis and multidisciplinary management of this potentially life-threatening complication.
